# Just‐in‐time display of minor red blood cell antibodies

**DOI:** 10.1111/trf.18459

**Published:** 2025-10-29

**Authors:** Michael Pettit, Randy Winstead, Kaycie Atchison, Angela Mueller, Jennifer Andrews

**Affiliations:** ^1^ Department of Pathology Vanderbilt University Medical Center Nashville Tennessee USA; ^2^ Health IT Vanderbilt University Medical Center Nashville Tennessee USA; ^3^ Quality, Safety & Risk Prevention Vanderbilt University Medical Center Nashville Tennessee USA; ^4^ Blood Bank Vanderbilt University Medical Center Nashville Tennessee USA; ^5^ Departments of Pathology & Pediatrics Vanderbilt University Medical Center Nashville Tennessee USA

**Abbreviations**
EHR: electronic health record
RBC: red blood cell

## 1 INTRODUCTION

The US healthcare system is responsible for nearly 11 million transfusions per year.^1^ Standardized pretransfusion testing helps mitigate the risks of this common intervention; however, antibody evanescence and the lack of immunohematology education pose difficulties for clinical teams.^2^, ^3^, ^4^, ^5^

Previously, our institution implemented an EHR “transfusion dashboard” and a “blood tracker” available in each individual patient chart, aiding clinicians in blood product ordering.^6^, ^7^ To further these efforts, we have implemented an EHR antibody alert system that organizes current and historical antibody data from our laboratory information system only in 3 different risk stratifications (see Table 1) with corresponding recommendations.

**TABLE 1:** Criteria for the assignment of three different levels of antibody alerts. The three tiers are “easy,” “medium,” and “difficult” and are determined by the results of the antibody ID work‐up, as seen on the right side of the table.

Risk stratification	Antibodies
“Easy”(one of the following)	Anti‐D, ‐C, ‐E, ‐K, ‐Le^a^, Le^b^, Lu^a^, ‐M, ‐N, non‐specific, passive anti‐D, panagglutinin
“Medium”	Two of the antibodies from the “easy” risk stratification, *or* one of the following: anti‐c, ‐e, ‐Fy^a^, ‐Fy^b^, ‐Jk^a^, ‐Jk^b^, ‐S, ‐s
“Difficult”	Three antibodies from any of the above, *or* one of the following: anti‐U, ‐Vel, ‐Di^a^, ‐Jk3

## 2 “EASY” ALLOIMMUNIZED PATIENT

**FIGURE 1:**
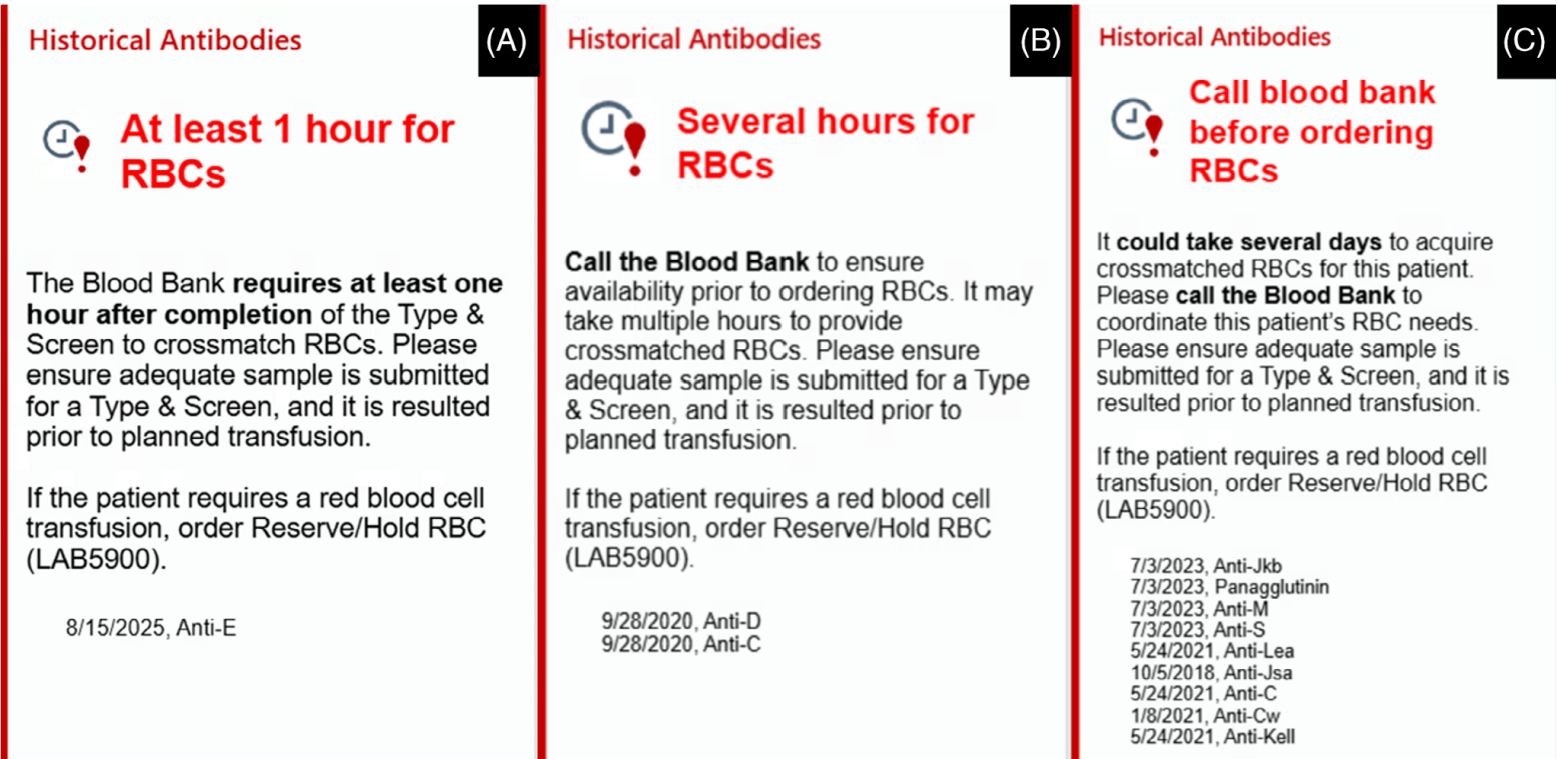
Historical antibodies.

The patient is a 39‐year‐old multiparous female in her second trimester without significant history with an anti‐E. See the EHR alert message (Figure 1A).

## 3 “MEDIUM” ALLOIMMUNIZED PATIENT

The patient is a 79‐year‐old female with a history of hypertension and colon cancer with anti‐D and anti‐C. See the EHR alert message (Figure 1B).

## 4 “DIFFICULT” ALLOIMMUNIZED PATIENT

The patient is a 27‐year‐old female with a history of sickle cell disease (HbSS), with many complications with a panagglutinin, anti‐Jkb, anti‐M, anti‐S, anti‐Lea, anti‐Jsa, anti‐C, anti‐Cw, and anti‐Kell. See the EHR alert message (Figure 1C).

## 5 CONCLUSION

We implemented an EHR antibody alert system that has successfully resulted in 850 reserve/hold RBC orders from July 1, 2024, to June 30, 2025, the recommended action of this alert.

## CONFLICT OF INTEREST STATEMENT

The authors declare no conflicts of interest.

## Data Availability

The data that support the findings of this study are available on request from the corresponding author. The data are not publicly available due to privacy or ethical restrictions.
